# Double reinforcement learning for cluster synchronization of Boolean control networks under denial of service attacks

**DOI:** 10.1371/journal.pone.0327252

**Published:** 2025-07-03

**Authors:** Wanqiu Deng, Chi Huang, Qinghong Shuai

**Affiliations:** 1 School of Management Science and Engineering, Southwestern University of Finance and Economics, Chengdu, China; 2 School of Computing and Artificial Intelligence, Southwestern University of Finance and Economics, Chengdu, China; 3 Engineering Research Center of Intelligent Finance, Ministry of Education, Southwestern University of Finance and Economics, Chengdu, China; University of Milano–Bicocca: Universita degli Studi di Milano-Bicocca, ITALY

## Abstract

This paper investigates the asymptotic cluster synchronization of Boolean control networks (BCNs) under denial-of-service (DoS) attacks, where each state node in the network experiences random data loss following a Bernoulli distribution. First, the algebraic representation of BCNs under DoS attacks is established using the semi-tensor product (STP) of matrices. Using matrix-based methods, some necessary and sufficient algebraic conditions for BCNs to achieve asymptotic cluster synchronization under DoS attacks are derived. For both model-based and model-free cases, appropriate state feedback controllers guaranteeing asymptotic cluster synchronization of BCNs are obtained through set-iteration and double-deep Q-network (DDQN) methods, respectively. Besides, a double reinforcement learning algorithm is designed to identify suitable state feedback controllers. Finally, a numerical example is provided to demonstrate the effectiveness of the proposed approach.

## Introduction

Boolean Networks (BNs), first introduced by Kauffman in 1969 as fundamental computational models for gene regulatory networks [1], have evolved into a versatile paradigm for analyzing complex dynamical systems. By abstracting system components as binary-state nodes (“on"/“off") governed by logical interaction rules, BNs achieve remarkable balance between computational tractability and biological plausibility [2,3]. This unique characteristic has fueled their widespread adoption across diverse domains, from cellular differentiation modeling [4,5] to power grid stability analysis [6]. The subsequent development of Boolean control networks (BCNs) by Akutsu *et al*. [7] introduced external control inputs, creating a powerful framework for studying targeted intervention strategies in networked systems. Recent advances in semi-tensor product (STP) theory [8] have further propelled BCNs research by enabling rigorous algebraic treatment of logical dynamics [9–11], as evidenced by emerging applications in smart grids [6], filter design [12] and multi-agent systems [13].

Due to their simple yet efficient modeling characteristics in gene regulatory networks [1,7], BNs and BCNs have been widely applied in real-world applications such as smart healthcare, intelligent home automation, smart transportation, and robotics. Particularly, therapeutic interventions for Parkinson’s disease [14], mathematical formulation of context-aware systems [15], optimal control design for urban traffic flow management [16], and robotic control architectures [17] have demonstrated based on BNs or/and BCNs methods. Moreover, in recent years, the financial domain has emerged as a particularly compelling application scenario for BNs and BCNs modeling [18]. Modern financial ecosystems comprise intricately interconnected entities—banks, investment firms, clearinghouses, and digital platforms—that exhibit nonlinear interdependencies akin to biological networks [19]. Network-based analyses have successfully captured phenomena like risk contagion in interbank markets [20] and crisis propagation dynamics [21]. However, current approaches predominantly employ continuous-variable models that may obscure essential discrete decision-making processes. This gap motivates our investigation of BCNs as a novel modeling paradigm for financial networks, particularly given their proven capacity to capture threshold-driven behaviors and abrupt state transitions characteristic of financial crises [22].

Denial-of-service (DoS) attacks represent a persistent and challenging threat in signal transmission processes. It is well-established that insufficient bit rates in communication channels degrade the stability of networked control systems [23], including BNs [24]. DoS attacks exploit network vulnerabilities to disrupt service availability by exhausting computational or bandwidth resources, leading to unauthorized or accidental data alteration, destruction, or loss [25]. These attacks are pervasive across many critical domains, including power grids [26], transportation networks [27], and financial networks [28]. Particularly in financial networks, the risks are exacerbated by their reliance on time-sensitive operations and the cascading effects of synchronization failures—a phenomenon in which clustered entities adopt coordinated strategies essential for maintaining market stability [29,30]. The disruption of such synchronization mechanisms during DoS events can trigger systemic failures through misaligned risk assessments and liquidity mismatches [31].

This paper addresses two fundamental challenges in securing financial BCNs under DoS attacks: (1) ensuring asymptotic cluster synchronization with known network topologies, and (2) achieving equivalent synchronization guarantees when node interaction rules are partially observable. While STP-based methods have demonstrated effectiveness for structured BCN analysis [32], the opacity of real-world financial networks necessitates innovative data-driven approaches. Recent breakthroughs in reinforcement learning (RL), particularly double deep Q-networks (DDQN) [33], offer promising solutions for control synthesis in partially observable environments [34]. However, existing RL applications to BCNs [35] have not adequately addressed the unique temporal constraints and attack resilience requirements of financial systems.

Our principal contributions are threefold:

(1) We establish a novel STP-based representation for BCNs under DoS attacks, explicitly characterizing state transitions through attack-dependent matrix operations. This formalism extends conventional BCN models by incorporating time-varying attack impact matrices.
(2) For systems with known topologies, we derive necessary and sufficient matrix conditions for asymptotic cluster synchronization using set-iteration methods. The proposed state feedback controller guarantees synchronization within finite time steps, even under intermittent DoS disruptions.
(3) Addressing scenarios with unknown node interactions, we develop a dual RL architecture combining model-based policy iteration with DDQN-based exploration. This hybrid approach efficiently discovers stabilizing controllers without requiring prior knowledge of logical rules, significantly expanding BCN applicability to opaque financial networks.

The remainder of this paper is organized as follows: Section II reviews STP fundamentals and formulates the synchronization problem. Sections III details our controller design methodologies for known and unknown network structures, respectively. Section IV validates the framework through financial network simulations, followed by concluding remarks in Section V.

*Notations:* 𝒟={0,1}. ℕ (${\mathbb{N}}^{+}$) is the set of non-negative (positive) integers. [a:b] is the set of all integers *l* satisfying a≤l≤b. The set of n×m real (column stochastic) matrices is represented by $R_{n\times m}$ ($P_{n\times m}$). ${\text{Col}}_{i}(M)$ $\left({\text{Row}}_{i}(M)\right)$ denotes the *i*th column (row) of the matrix *M*. For any $L\in R_{n\times m}$, *L* is an n×m logical matrix if ${\text{Col}}_{i}(L)\in \Delta_{n}$ for all i∈[1:m]. $L_{n\times m}$ is the set of n×m logical matrices. *I*_*n*_ represents the n×n identity matrix. $\Delta_{n}:=\{\delta_{n}^{i}|i\in [1:n]\}$, where $\delta_{n}^{i}$denotes the *i*th column of the identity matrix *I*_*n*_. A matrix *M* = $\left[\delta_{m}^{i_{1}}\;\delta_{m}^{i_{2}}\;\cdots \;\delta_{m}^{i_{n}}\right]$ can be simply denoted by $M=\delta_{m}[i_{1}\;i_{2}\;\cdots \;i_{n}]$. *[M]*_*ij*_ represents the (*i*,*j*)th entry of the matrix *M*. $D_{1}=\delta_{2}[1\;1\;2\;2]$ and $D_{2}=\delta_{2}[1\;2\;1\;2]$ called the dummy matrices. $W_{\left[m,n\right]}:=[I_{n}⊗\delta_{m}^{1}\;\cdots \;I_{n}⊗\delta_{m}^{m}]$ is the mn×mn swap matrix, where ⊗ is Kronecker product. $\Phi_{n}=\delta_{2^{2n}}[1,2^{n}\;+\;2,\cdots ,(2^{n}\;-\;2)\times 2^{n}+2^{n}-1,2^{2n}]$ is the power-reducing matrix. |𝒜| represents the cardinal number of the set 𝒜. Pr{A} is the probability of an event *A*; Pr{A|B} is the conditional probability of an event *A* given that an event *B* has occurred. For two sets $𝒳=\{x_{i}\in \Delta_{n}\}$ and $𝒴=\{y_{j}\in \Delta_{m}\}$, define $𝒳⋉𝒴=\{x_{i}⋉y_{j}\in \Delta_{n+m}|x_{i}\in 𝒳,y_{j}\in 𝒴\}$.

## Preliminaries and problem formulations

In this section, some necessary preliminaries are presented, including an introduction to the STP method, formulations of a BCN under DoS attacks, and a formal statement of asymptotic cluster synchronization.

### STP preliminaries

First, let $A\in R_{m\times s},B\in R_{r\times q}$, the Kronecker product [36] of *A* and *B* is defined as follows:


$$A⊗B=\left(\begin{array}{cccc} a_{11}B & a_{12}B & \cdots & a_{1s}B\\ a_{21}B & a_{22}B & \cdots & a_{2s}B\\ \vdots & \vdots & \ddots & \vdots \\ a_{m1}B & a_{m2}B & \cdots & a_{ms}B \end{array}\right).$$

Besides, for two matrices $A=[{𝐚}_{1}\;{𝐚}_{2}\;\cdots \;{𝐚}_{s}]\in R_{m\times s}$ and $C=[{𝐜}_{1}\;{𝐜}_{2}\;\cdots \;{𝐜}_{s}]\in R_{p\times s}$, the Khatri-Rao product of *A* and *B* is defined as $A*B=[{𝐚}_{1}⊗{𝐜}_{1}\;{𝐚}_{2}⊗{𝐜}_{2}\cdots {𝐚}_{s}⊗{𝐜}_{s}]\in R_{(m+p)\times s}$, where ${𝐚}_{i}\in R_{m\times 1}$ and ${𝐜}_{i}\in R_{p\times 1}$, i=1,2,…,s.

Then, the definition of the STP of matrices is presented as follows:

**Definition 1** ([8]). *Consider two real matrices $M\in R_{m\times n}$ and $N\in R_{p\times q}$, their STP M⋉N is defined as follows:*


$$M⋉N=(M⊗I_{\alpha /n})(N⊗I_{\alpha /p}),$$

*where α=lcm(n,p) is the least common multiple of n and p, and ⊗ is the Kronecker product.*

Note that M⋉N=M×N if *q* = *n* in Definition (1), where × is the conventional matrix multiplication. Due to this dimensional compatibility, the symbol ⋉ will be omitted in subsequent discussions for notational simplicity when no ambiguity arises. The STP method has achieved a breakthrough in the dimensionality of matrix multiplication, thereby enabling the establishment of an equivalent algebraic form of logical functions, as demonstrated by the following lemma.

**Lema 1** ([8]). *Given a logical function $(y_{1},...,y_{m})=f(x_{1},...,x_{n}):D^{n}\to D^{m}$, there is a unique matrix which is called the structural matrix $F\in L_{2^{m}\times 2^{n}}$ such that*


$${⋉}_{j=1}^{m}Y_{i}=F{⋉}_{i=1}^{n}X_{i},$$

*where $X_{i}=\left[\begin{array}{c} x_{i}\\ \neg x_{i} \end{array}\right]=\delta_{2}^{2-x_{i}},Y_{j}=\left[\begin{array}{c} y_{i}\\ \neg y_{i} \end{array}\right]=\delta_{2}^{2-y_{i}}\in \Delta_{2}$ are the vector form of logic variables $x_{i},y_{j}\in D$, respectively, i∈[1:n], j∈[1:m]*.

### Model descriptions

A BCN with DoS attacks, comprising *n* state nodes and *m* control inputs, is mathematically described as follows:

$$x_{i}(t+1)=f_{i}(y_{1}(t),\ldots ,y_{n}(t),u_{1}(t),\ldots ,u_{m}(t)),i\in [1:n],t\in \mathbb{N}.$$ (1)

Here, logical variables $y_{i}(t)\in D$ and $x_{i}(t)\in D$ represent the received data and actual data of node *i* at time $t\in {\mathbb{N}}^{+}$, respectively. $f_{i}:D^{n+m}\to D$ is a logical function, i∈[1:n]. Besides, the logical variable $u_{j}(t)\in D$ denotes the control input at time *t*, where its logical relationship with the states is expressed as follows:

$$u_{j}(t)=g_{j}(x_{1}(t),\ldots ,x_{n}(t),y_{1}(t-1),\ldots ,y_{m}(t-1)),j\in [1:m],t\in \mathbb{N}.$$ (2)

Here, $g_{j}:D^{2n}\to D$ is a logical function, j∈[1:m]. Besides, it should be noted that *y*_*i*_(−1) is a predetermined value. Since this paper focuses on the analysis of global cluster synchronization, *y*_*i*_(−1) arbitrarily chosen from the set 𝒟 for all i∈[1:n].

To characterize the impact of DoS attacks on the data received by system (1), the Bernoulli distribution is used. The data transmission process for each node of (1) can be described as follows:

$$y_{i}(t)=\mu_{i}(t)x_{i}(t)+[1-\mu_{i}(t)]y_{i}(t-1),$$ (3)

where i∈[1:n] and t∈ℕ. Given a subset 𝒞⊆[1:n], called a constrained set of the system (1), and for all i∈𝒞, the sequence $\mu_{i}(t)$ is modeled as a Bernoulli distributed random variable with the following probability distribution:

$$\mathrm{Pr}\{\mu_{i}(t)=1\}=a_{i},\;\mathrm{Pr}\{\mu_{i}(t)=0\}=1-a_{i},$$ (4)

where $a_{i}\in R$ and satisfies $0<a_{i}\leq 1$, and the random variables $\mu_{i}(t)$ are assumed to be mutually independent for all i∈[1:n]. One can obtain that the data of *x*_*i*_ has been successfully transmitted at time *t* when $\mu_{i}(t)=1$. Otherwise, $\mu_{i}(t)=0$ indicates that its data has been lost, and the latest received data will be used as a substitution. In addition, due to limited resources and the design of defensive measures, DoS attacks do not target all nodes in the network [37–40]. Therefore, the following set is defined as 𝒱:=[1:n]\𝒞, and for all l∈𝒱, the sequence $\mu_{l}(t)$ satisfies

$$\mathrm{Pr}\{\mu_{l}(t)=1\}=1.$$ (5)

This indicates that the nodes of (1) in 𝒱 are not affected by DoS attacks.

Let binary values 1 and 0 as the vectors $\delta_{2}^{1}$ and $\delta_{2}^{2}$, respectively. Then, consider system (1), let $X_{i}(t)=\delta_{2}^{2-x_{i}(t)}$, $Y_{i}(t)=\delta_{2}^{2-y_{i}(t)}$, and $U_{i}(t)=\delta_{2}^{2-u_{i}(t)}$ denote the vector forms of $x_{i}(t),\;y_{i}(t)$, and *u*_*i*_(*t*) at time *t*, respectively. In addition, let $X(t)={⋉}_{i=1}^{n}X_{i}(t)\in \Delta_{2^{n}}$, $Y(t)={⋉}_{i=1}^{n}Y_{i}(t)\in \Delta_{2^{n}}$, and $U(t)={⋉}_{i=1}^{m}U_{i}(t)\in \Delta_{2^{m}}$. By utilizing Lemma 1, system (1) can be converted into the following equivalent algebraic form,

X(t+1)=F⋉U(t)⋉Y(t), (6)

where $F=F_{1}*F_{2}*\ldots *F_{n}\in L_{2^{n}\times 2^{n+m}}$, and * is the Khatri-Rao product. Here, $F_{i}\in L_{2^{\times }2^{n+m}}$ is the structure matrix of the logical function *f*_*i*_ in system (1), i∈[1:n]. In addition, control (2) can be converted into the following equivalent algebraic form,

U(t)=KX(t)Y(t−1). (7)

It is the state feedback control of system (6), where $K=G_{1}*G_{2}*\ldots *G_{n}\in L_{2^{m}\times 2^{2n}}$ is the state feedback gain matrix of (6). Here, $G_{j}\in L_{2\times 2^{2n}}$ is the structure matrix of the logical function *g*_*i*_ in control (2), j∈[1:m].

### Problem formulations

Consider system (6), the transition of X(t+1) is made to depend stochastically on Y(t), thus forming a non-iterative system. Therefore, in order to convert system (6) to iterative form, it is necessary to analyse the properties of *Y*(*t*). First, assume that constrained set $𝒞=\{i_{1},i_{2},\ldots ,i_{s}\}\subseteq [1:n]$, where $i_{l_{1}}<i_{l_{2}}$ if $l_{1}<l_{2}$, then based on the construction of swap matrix, one has ${⋉}_{k=0}^{s-1}W_{\left[2^{i_{s-k}-1+k},2\right]}{⋉}_{j=1}^{n}X_{j}={⋉}_{l=1}^{s}X_{i_{j}}X_{1}\cdots X_{i_{1}-1}X_{i_{1}+1}\cdots X_{n}$. Therefore, for simplicity, let constrained set 𝒞=[1:s] in (6), *s* < *n*, then *Y*(*t*) can be expressed as follows:


$$Y(t)={⋉}_{i=1}^{s}Y_{i}(t){⋉}_{l=s+1}^{n}X_{l}(t)$$

$$={⋉}_{i=1}^{s}\left[\eta_{i}(t)X_{i}(t)+(1-\eta_{i}(t))Y_{i}(t-1)\right]{⋉}_{l=s+1}^{n}X_{l}(t)$$

$$=\sum_{i_{1}=1}^{2}...\sum_{i_{s}=1}^{2}\prod_{j=1}^{s}\gamma_{i_{j}}(t){⋉}_{j=1}^{s}\left(D_{i_{j}}X_{j}(t)Y_{j}(t-1)\right){⋉}_{l=s+1}^{n}X_{l}(t)$$

$$=\sum_{i_{1}=1}^{2}...\sum_{i_{s}=1}^{2}\prod_{j=1}^{s}\gamma_{i_{j}}(t)\left({⋉}_{i=1}^{s}(I_{2^{2(j-1)}}⊗D_{i_{j}})\right)\left({⋉}_{j=1}^{s}X_{j}(t)Y_{j}(t-1)\right){⋉}_{l=s+1}^{n}X_{l}(t)$$

$$=\sum_{i_{1}=1}^{2}...\sum_{i_{s}=1}^{2}\prod_{j=1}^{s}\gamma_{i_{j}}(t)\left({⋉}_{i=1}^{s}(I_{2^{2(j-1)}}⊗D_{i_{j}})\right)$$

$$\left({⋉}_{j=1}^{s-1}I_{2^{j}}⊗W_{\left[2,2^{j}\right]}\right)\left(I_{2^{s}}⊗W_{\left[2^{n-s},2^{s}\right]}\right)\left(I_{2^{n+s}}⊗1_{2^{n-s}}\right)X(t)Y(t-1)$$

$$≜\sum_{c=1}^{2^{s}}\tau_{c(i_{1},...,i_{s})}\times D_{c(i_{1},...,i_{s})}ℳX(t)Y(t-1),$$ (8)

where $\gamma_{i_{j}}=\left\{ \begin{array}{c} \eta_{j},\;\;\;\;\;\;i_{j}=1\\ 1-\eta_{j},\;i_{j}=2 \end{array}\right.$, $\tau_{c(i_{1},...,i_{n})}=\prod_{j=1}^{n}\gamma_{i_{j}}$, $D_{i_{j}}$ = $\left\{ \begin{array}{c} D_{1},\;i_{j}=1\\ D_{2},\;i_{j}=2 \end{array}\right.$, $D_{c(i_{1},...,i_{s})}={⋉}_{j=1}^{s}(I_{2^{2(s-j)}}⊗D_{i_{j}})$, $c_{\left(i_{1},...,i_{s}\right)}=\sum_{j=1}^{s}(i_{j}\;-\;1)\;\times \;2^{j-1}\;+\;1$, and $ℳ=\left({⋉}_{j=1}^{s-1}I_{2^{j}}⊗W_{\left[2,2^{j}\right]}\right)\left(I_{2^{s}}⊗W_{\left[2^{n-s},2^{s}\right]}\right)\left(I_{2^{n+s}}⊗1_{2^{n-s}}\right)$. For the sake of convenience, the symbol *c* is utilized to represent $c_{\left(i_{1},...,i_{s}\right)}$ in the rest of this paper, without repetition. It is easy to see that the function *c* has a range from 1 to $2^{\text{s}}$.

To address asymptotic cluster synchronization in BCNs under DoS attacks, define Z(t)=X(t)Y(t−1), then the following augmented system is obtained. The relationship between this augmented system and the original system (6) will be discussed in Lemma 2 and Section III.


$$𝔼\{Z(t+1)\}=𝔼\{X(t+1)Y(t)\}=FU(t)\Phi_{n}𝔼\{Y(t)\}$$

$$=\sum_{c=1}^{2^{s}}\tau_{c}\times F(I_{2^{m}}⊗\Phi_{n})(I_{2^{m}}⊗D_{c}ℳ)U(t)𝔼\{Z(t)\}$$

$$=\sum_{c=1}^{2^{s}}\tau_{c}\times L_{c}U(t)𝔼\{Z(t)\}≜𝐅U(t)𝔼\{Z(t)\},$$ (9)

where $𝐅=\sum_{c=1}^{2^{s}}\tau_{c}\times L_{c}\in P_{2^{2n}\times 2^{m+2n}}$, $L_{c}=F(I_{2^{m}}⊗\Phi_{n})(I_{2^{m}}⊗D_{c}ℳ)$, and $\tau_{c}$ is defined in (8). The following result will establish the equivalence between the asymptotic stability of systems (6) and (9).

**Lemma 2.** *Consider system (6) with a given target set $𝒳\subseteq \Delta_{2^{n}}$, the following two statements are equivalent.*

*(i) For any initial states $X_{0},Y_{-1}\in \Delta_{2^{n}}$ in system (6), there exists a control law $\pi_{u}:\Delta_{2^{2n}}\to \Delta_{2^{m}}$, which is given by U(t)=KX(t)Y(t−1), such that*
  $$\lim_{t\to \infty }\mathrm{Pr}\{X(t)\in 𝒳|X_{0},Y_{-1},\pi_{u}\}=1.$$ (10)
*(ii) For any initial state $Z_{0}=X_{0}\;⋉\;Y_{-1}\in \Delta_{2^{2n}}$ in system (9), there exists a control law $\pi_{u}:\Delta_{2^{2n}}\to \Delta_{2^{m}}$, which is given by U(t)=KX(t)Y(t−1), such that*
  $$\lim_{t\to \infty }\mathrm{Pr}\{Z(t)\in 𝒵|Z_{0},\pi_{u}\}=1.$$ (11)
*Here, $𝒵=𝒳⋉𝒳\subseteq \Delta_{2^{2n}}$.*

*Proof:* (Necessity) Consider a given target set $𝐗\in \Delta_{2^{n}}$, for any initial states $X_{0},Y_{-1}\in \Delta_{2^{n}}$, the condition $\lim_{t\to \infty }\mathrm{Pr}\{X(t)\in X|X_{0},Y_{-1},\pi_{u}\}=1$ implies that for any fixed ϵ>0, there always exists an integer *T* such that for any *t*>*T*, $P\{X(t)\in X|X_{0},Y_{-1},\pi_{u}\}>1\;-\;\frac{\epsilon }{2n}$ holds. Let $𝒵=𝒳⋉𝒳\subseteq \Delta_{2^{2n}}$, and assume that there exists an initial state $Z_{0}=X_{0}⋉Y_{-1}$, such that for any t∈ℕ, one can find a real number $\epsilon^{\prime }>0$ satisfying the following condition,


$$\mathrm{Pr}\{Z(t)\notin 𝒵|Z_{0},\pi_{u}\}\geq \frac{(2n+1)\epsilon^{\prime }}{2n}.$$

Then, one has that


$$\begin{array}{cc} & \mathrm{Pr}\{Z(t)\notin 𝒵|Z_{0},\pi_{u}\}=\mathrm{Pr}\{X(t)⋉Y(t-1)\notin 𝒵|X_{0},Y_{-1},\pi_{u}\}\\ & \leq \mathrm{Pr}\{X(t)\notin 𝒳|X_{0},Y_{-1},\pi_{u}\}+\mathrm{Pr}\{Y(t-1)\notin 𝒳|X_{0},Y_{-1},\pi_{u}\}. \end{array}$$

Furthermore, for the fixed real number $\epsilon^{\prime }>0$, one can find an integer $T^{\prime }$, such that $\mathrm{Pr}\{X(t)\notin 𝒳|X_{0},Y_{-1},\pi_{u}\}<\frac{\epsilon^{\prime }}{2n}$. Since


$$\frac{(2n+1)\epsilon^{\prime }}{2n}\leq \mathrm{Pr}\{Z(t)\notin 𝒵|Z_{0},\pi_{u}\}<\frac{\epsilon^{\prime }}{2n}+\mathrm{Pr}\{Y(t-1)\notin 𝒳|X_{0},Y_{-1},\pi_{u}\}.$$

Therefore, one has that

$$\mathrm{Pr}\{Y(t-1)\notin 𝒳|X_{0},Y_{-1},\pi_{u}\}>\epsilon^{\prime },$$ (12)

holds for all t∈ℕ. However, let


$$T_{i}=\left\{ \begin{array}{cc} T^{\prime }+1, & \text{if }a_{i}=1,\\ \lceil \log_{1-a_{i}}\frac{\epsilon^{\prime }}{2n}\rceil +T^{\prime }+1, & \text{if }a_{i}<1. \end{array}\right.$$

Based on $\mathrm{Pr}\{X(t)\in 𝒳|X_{0},Y_{-1},\pi_{u}\}>1-\frac{\epsilon^{\prime }}{2n}$, one has that


$$\&\mathrm{Pr}\{Y_{i}(t-1)\notin \psi_{i}(𝒳)|X_{0},Y_{-1},\pi_{u}\}\;=\&\mathrm{Pr}\{\mu_{i}(t-1)=1\}\times \mathrm{Pr}\{X_{i}(t-1)\notin \psi_{i}(𝒳)|X_{0},Y_{-1},\pi_{u}\}\;\&+\mathrm{Pr}\{\mu_{i}(t-1)=0\}\times \mathrm{Pr}\{Y_{i}(t-2)\notin \psi_{i}(𝒳)\}\;<\&\frac{a_{i}\epsilon^{\prime }}{2n}+(1-a_{i})\times \mathrm{Pr}\{Y_{i}(t-2)\notin \psi_{i}(𝒳)|X_{0},Y_{-1},\pi_{u}\}\;<\&\frac{a_{i}\epsilon^{\prime }}{2n}+\frac{a_{i}(1-a_{i})\epsilon^{\prime }}{2n}+\ldots +\frac{a_{i}(1-a_{i}{)}^{t-T^{\prime }-2}\epsilon^{\prime }}{2n}\;\&+(1-a_{i}{)}^{t-T^{\prime }-1}\times \mathrm{Pr}\{Y_{i}(T^{\prime })\notin \psi_{i}(𝒳)|X_{0},Y_{-1},\pi_{u}\}\;\leq \&\frac{\epsilon^{\prime }}{2n}[1-(1-a_{i}{)}^{t-T^{\prime }-1}]+(1-a_{i}{)}^{t-T^{\prime }-1}\;<\&\frac{\epsilon^{\prime }}{2n}+(1-a_{i}{)}^{T_{i}-T^{\prime }-1}<\frac{\epsilon^{\prime }}{n}.$$

Then, one can obtain that $\mathrm{Pr}\{Y(t\;-\;1)\notin 𝒳\}\leq \sum_{i=1}^{n}\mathrm{Pr}\{Y_{i}(t\;-\;1)\notin \psi_{i}(𝒳)\}<\epsilon^{\prime }$ if $t>\overset{―}{T}:=\max\{T_{1},\cdots ,T_{n}\}$. This contradicts (12). Therefore, for any state $Z_{0}\in \Delta_{2^{2n}}$, and any fixed ϵ>0, there exists an integer $\overset{―}{T}$ and a control law $\pi_{u}$, such that $\mathrm{Pr}\{Z(t)\notin 𝒵|Z_{0},\pi_{u}\}<\frac{(2n+1)\epsilon }{2n}$. Thus, (11) holds.

(Sufficiency) Since Z(t)∈𝒵 is equivalent to X(t)∈𝒳 and Y(t−1)∈𝒳, it implies that $\lim_{t\to \infty }\mathrm{Pr}\{Z(t)\in 𝒵|Z_{0},\pi_{u}\}\leq \lim_{t\to \infty }\mathrm{Pr}\{X(t)\in 𝒳|Z_{0},\pi_{u}\}$. It follows from $\lim_{t\to \infty }\mathrm{Pr}\{Z(t)\in 𝒵|Z_{0},\pi_{u}\}=1$ and the squeeze theorem that $\lim_{t\to \infty }\mathrm{Pr}\{X(t)\in 𝒳|Z_{0},\pi_{u}\}=1$. ◻

Next, the definition of asymptotic cluster synchronization with probability one for system (1) is expressed below.

**Definition 2** ([41]). *(CSPO) Consider system (1), whose state nodes can be divided into p clusters $\left\{\Theta_{1},\Theta_{2},\ldots ,\Theta_{p}\right\}$, such that $\sum_{i=1}^{p}\Theta_{i}=[1:n]$ and $\Theta_{i}\cap \Theta_{j}=\emptyset$ for i≠j. System (1) is said to achieve asymptotic cluster synchronization with probability one, if there exists a state feedback control law $\pi_{u}:{𝒟}^{2n}\to {𝒟}^{m}$, which is equivalent to U(t)=KX(t)Y(t−1), such that for each cluster $\Theta_{l}$, l∈[1:p], one has*

$$\lim_{t\to \infty }\mathrm{Pr}\{x_{i}(t)=x_{j}(t)|x_{0},y_{-1},\pi_{u}\}=1,\forall i,j\in \Theta_{l},\forall x_{0},y_{-1}\in {𝒟}^{n}.$$ (13)

*In addition, let $\Theta_{1}=\{1,2,\ldots ,k_{1}\},\ldots ,\Theta_{p}=\{k_{p-1}+1,k_{p-1}+2,\ldots ,k_{p}=n\}$.*

## Main results

In this section, a set-iteration method is first proposed to design the state feedback control (7) for achieving asymptotic cluster synchronization in system (1). For scenarios where the logical relationships between nodes are unknown in system (1), a DDQN algorithm is further developed to obtain the required state feedback control (7).

### Set-iteration method

First, a set is constructed in following, which is the target state set for achieving asymptotic cluster synchronization in system (1) [41],

$$ℋ=\{X|X=\delta_{2^{n}}^{(\sum_{i=1}^{p}(\beta_{i}-1)2^{n-k_{i}})+1},\beta_{i}=1,2^{k_{i}-k_{i-1}},i\in [1:m],\text{ and }k_{0}=0\}.$$ (14)

Then, in order to obtain equivalent algebraic conditions for asymptotic cluster synchronization of system (1), the following notions of control invariant subsets and largest control invariant subsets are given.

**Definition 3.** *Consider system (9), and given a set $𝒜\subseteq \Delta_{2^{2n}}$, a subset ℬ⊆𝒜 is a control invariant subset of 𝒜, if for any state Z∈𝒜, there exists a control input $U\in \Delta_{2^{m}}$, such that Pr{𝐅UZ∈ℬ}=1. In addition, the largest control invariant subset of 𝒜 is the union of all control invariant subsets contained in 𝒜, and denoted as $I_{c}(𝒜)$.*

**Theorem 1.** *System (1) achieves asymptotic cluster synchronization with probability one if and only if the following matrix equation has a solution,*

$$\text{sgn}\left[\sum_{\delta_{2^{2n}}^{i}\in [I_{c}(ℋ){]}^{2}}{\text{Row}}_{i}\left(\Gamma^{T}\right)\right]=1_{2^{n}}^{⊤}.$$ (15)

*Here, $\Gamma =𝐅K\Phi_{2n}$, matrix $K\in L_{2^{m}\times 2^{2n}}$ is the state feedback gain matrix of system (9), which is unknown, and* **F** *is defined in (9). $T=2^{2n}\;-\;|[I_{c}(ℋ){]}^{2}|$, where ℋ is defined in (14), $\left[I_{c}(ℋ){]}^{2}=I_{c}(ℋ)⋉I_{c}(ℋ)=\{X⋉Y\in \Delta_{2^{2n}}|X,Y\in I_{c}(ℋ)\right\}$, and sgn[·] denotes the sign function.*

Proof: According to the equivalent algebraic form (6) of system (1), for any initial states $x_{0},y_{-1}\in {𝒟}^{n}$ and feedback control law $\pi_{u}$, one has that $\mathrm{Pr}\{x_{i}(t)=x_{j}(t)|x_{0},y_{-1},\pi_{u}\}=\mathrm{Pr}\{X_{i}(t)=X_{j}(t)|X_{0},Y_{-1},\pi_{u}\}$ holds for all i,j∈[1:n] and t∈ℕ. Therefore, system (1) achieves asymptotic cluster synchronization with probability one if and only if there exists a state feedback control law $\pi_{u}$, such that for each cluster $\Theta_{l}$, l∈[1:p],

$$\lim_{t\to \infty }\mathrm{Pr}\{X_{i}(t)=X_{j}(t)|X_{0},Y_{-1},\pi_{u}\}=1,\forall i,j\in \Theta_{l},\forall X_{0},Y_{-1}\in \Delta_{2^{n}},$$ (16)

holds for system (6).

(Necessity) First, we proof that system (6) satisfies condition (10) with $𝒳=I_{c}(ℋ)$ if (16) holds. We employ a proof by contradiction to demonstrate this fact. Assume that there exists an initial state of system (6) $Z_{0}=X_{0}⋉Y_{-1}\in \Delta_{2^{2n}}$, such that for any state feedback control law $\pi_{u}$ and any time *T*_1_, there exists *t*>*T*_1_, one has that $\mathrm{Pr}\{X(t)\in I_{c}(ℋ)|Z_{0},\pi_{u}\}<1$. Then, there exists ϵ>0, such that $\mathrm{Pr}\{X(t)\notin I_{c}(H)|Z_{0},\pi_{u}\}>\epsilon$. According to the definition of largest control invariant subset, one can find a real number $\epsilon^{\prime }$ such that $\mathrm{Pr}\{X(t\;+\;1)\notin ℋ|Z_{0},\pi_{u}\}>\epsilon^{\prime }$. Let $X(t\;+\;1)={⋉}_{k=1}^{n}X_{k}(t\;+\;1)={⋉}_{k=1}^{n}\delta_{2}^{\alpha_{k}}$. Based on the construction of the set ℋ, there exist l∈[1:p], and $i,j\in \Theta_{l}$, such that $\mathrm{Pr}\{X_{i}(t+1)=\alpha_{i}\neq X_{j}(t+1)=\alpha_{j}|Z_{0},\pi_{u}\}>\epsilon^{\prime }$, which contradicts (16). Therefore, system (6) satisfies condition (10) with $𝒳=I_{c}(ℋ)$. Based on Lemma 2, system (6) satisfies condition (11) with $𝒳=I_{c}(ℋ)$. Consider the state feedback control law $\pi_{u}:U(t)=K^{\prime }Z(t)$ in system (6), which satisfies condition (11). Then, according to [42], $K^{\prime }$ is a solution to (17).

(Sufficiency) Assume that (17) has a solution, denoted by $K^{\prime }$. Then, according to cite [42], system (6) satisfies condition (11) with $𝒳=I_{c}(ℋ)$. Furthermore, based on Lemma (2), system (6) satisfies condition (10) with $𝒳=I_{c}(ℋ)$. According to the construction of the set ℋ, system (1) achieves asymptotic cluster synchronization with probability one. The detailed proof can be found in [41] and is omitted here. ◻

Let ${𝒲}_{ℋ}=I_{c}([I_{c}(ℋ){]}^{2})$, one has the following result, which proof is similar to that of [42] and is omitted here.

**Theorem 2.** *System (1) achieves asymptotic cluster synchronization with probability one if and only if the following matrix equation has a solution,*

$$\text{sgn}\left[\sum_{\delta_{2^{2n}}^{i}\in {𝒲}_{ℋ}}{\text{Row}}_{i}\left(\Gamma^{T}\right)\right]=1_{2^{n}}^{⊤}.$$ (17)

*Here, $\Gamma =𝐅K\Phi_{2n}$, matrix $K\in L_{2^{m}\times 2^{2n}}$ is the state feedback gain matrix of system (9), which is unknown, and* **F** *is defined in (9). $T=2^{2n}-|{𝒲}_{ℋ}|$, where ℋ is defined in (14), and sgn[·] denotes the sign function.*

Based on Theorem 2, the following easily verifiable sufficient criterion can be derived to check asymptotic cluster synchronization with probability one for system (1).

**Corollary 1.** *System (1) cannot achieve asymptotic cluster synchronization with probability one if (17) does not hold with respect to $K=1_{2^{m}\times 2^{2n}}$.*

*Proof:* Assume that (17) does not hold with respect to $K=1_{2^{m}\times 2^{2n}}$, however system (1) achieve asymptotic cluster synchronization with probability one. Based on Theorem 2, one has that there exist a feedback gian matrix $\overset{―}{K}\in L_{2^{m}\times 2^{2n}}$ such that for any initial state $Z_{0}\in \Delta_{2^{2n}}$, one has that $\mathrm{Pr}\{Z(2^{2n}-|{𝒲}_{ℋ}|;Z_{0},\pi_{u})\in {𝒲}_{ℋ}\}>0$. Thus, $\text{sgn}\left[\sum_{\delta_{2^{2n}}^{i}\in {𝒲}_{ℋ}}{\text{Row}}_{i}\left((𝐅\overset{―}{K}\Phi_{2n}{)}^{T}\right)\right]=1_{2^{n}}^{⊤}$, where $T=2^{2n}\;-\;|{𝒲}_{ℋ}|$. Therefore, $\text{sgn}\left[\sum_{\delta_{2^{2n}}^{i}\in {𝒲}_{ℋ}}{\text{Row}}_{i}\left((𝐅1_{2^{m}\times 2^{2n}}\Phi_{2n}{)}^{T}\right)\right]\geq \text{sgn}\left[\sum_{\delta_{2^{2n}}^{i}\in {𝒲}_{ℋ}}{\text{Row}}_{i}\left((𝐅\overset{―}{K}\Phi_{2n}{)}^{T}\right)\right]=1_{2^{n}}^{⊤}$, where $T=2^{2n}\;-\;|{𝒲}_{ℋ}|$. This demonstrates the validity of condition (17) with respect to $K=1_{2^{m}\times 2^{2n}}$, which necessarily induces a contradiction. ◻

However, solving equation (17) is challenging and often results in high computational complexity. Therefore, a set-iteration method is proposed to find a suitable matrix that satisfy equation (17).

Let $\Xi_{0}={𝒲}_{ℋ}$, where ℋ is defined in (14), and define $\Xi_{k+1}$ as follows:

$$\Xi_{k+1}=\left\{\delta_{2^{2n}}^{j}|\sum_{i\in \Xi_{k}}\left[{\left(\delta_{2^{2n}}^{i}\right)}^{⊤}\left(\sum_{l=1}^{2^{m}}{𝐅}_{l}\right)\delta_{2^{2n}}^{j}\right]>0\right\},k\in \mathbb{N}.$$ (18)

The set $\Xi_{k}$, k∈ℕ, constructed through the iterative process defined by (18), represents the states reachable from ${𝒲}_{ℋ}$ in at most *k* steps. Based on this construction, the following result holds for all k∈ℕ.

**Theorem 3.** *System (1) achieves asymptotic cluster synchronization with probability one if and only if the following conditions hold for system (9),*

*(i) set $\Xi_{0}$ is nonempty;*
*(ii) there exists a positive integer $T\leq 2^{2n}-|{𝒲}_{ℋ}|$, such that $\Xi_{T}=\Delta_{2^{2n}}$.*

*Proof:* (Necessity) As systems (1) and (6) are equivalent, it follows that system (6) achieves asymptotic cluster synchronization with probability one. Then, condition (i) is obvious by the construction of the set ℋ. According to Theorem 2, equation (17) has a solution, and denoted by *K*. Furthermore, due to the existence of *K* of (17), for any initial state $Z_{0}=\delta_{2^{2n}}^{i_{0}}$ of system (9), there exists a trajectory of *Z*_0_ given by


$$Z_{0}=\delta_{2^{2n}}^{i_{0}}\to \delta_{2^{2n}}^{i_{1}}\to \cdots \to \delta_{2^{2n}}^{i_{k(Z_{0})}}\in {𝒲}_{ℋ}.$$

Here, for any state $\delta_{2^{2n}}^{i_{s}}$, $s\in [1:k(Z_{0})-1]$, there always exists a control $\delta_{2^{m}}^{l_{s}}\in \Delta_{2^{m}}$ such that $[F_{l_{s}}{]}_{i_{s+1},i_{s}}>0$, where $\delta_{2^{m}}^{l_{s}}={\text{Col}}_{i_{s}}(K)$. Thus, one has that $Z_{0}\in \Xi_{k(Z_{0})}$.

Next, we use the converse to show that $k(Z_{0})\leq 2^{2n}\;-\;|{𝒲}_{ℋ}|$ for all initial state *Z*_0_. Assume that there exists an initial state ${\text{Z}}_{0}$ such that ${\text{Z}}_{0}\in \Xi_{k^{\prime }({\text{Z}}_{0})}$, where $k^{\prime }({\text{Z}}_{0})>2^{2n}\;-\;|[I_{c}(ℋ){]}^{2}|$. In addition, construct a following trajectory, which start from ${\text{Z}}_{0}$ to $I_{c}(ℋ)$,

$${\text{Z}}_{0}=\delta_{2^{2n}}^{i_{0}}\to \cdots \to \delta_{2^{2n}}^{i_{p}}\to \cdots \to \delta_{2^{2n}}^{i_{p+q}}\to \cdots \to \delta_{2^{2n}}^{i_{k^{\prime }({\text{Z}}_{0})}}\in {𝒲}_{ℋ}.$$ (19)

Since $\left|\Delta_{2^{2n}}\backslash {𝒲}_{ℋ}|<2^{2n}\;-\;|{𝒲}_{ℋ}\right|$, there exist two integers $i_{p},i_{p+q}\in [1:k^{\prime }({\text{Z}}_{0})\;-\;1]$ in the trajectory (19), such that $\delta_{2^{2n}}^{i_{p}}=\delta_{2^{2n}}^{i_{p+q}}$, where $q>k^{\prime }({\text{Z}}_{0})\;-\;|{𝒲}_{ℋ}|$. Furthermore, one has that ${\text{Col}}_{i_{p}}(K)\neq Col_{i_{p+q}}(K)$, Then, a contradiction arises since $K\in L_{2^{m}\times 2^{2n}}$. Therefore, there exists an integer $k({\text{Z}}_{0})=k({\text{Z}}_{0}^{\prime })-q<2^{2n}-|{𝒲}_{ℋ}|$, such that ${\text{Z}}_{0}\in \Xi_{k({\text{Z}}_{0})}$.

(Sufficiency) Assume that for any $Z_{0}\in \Delta_{2^{2n}}$, there exists an integer $k(Z_{0})<2^{2n}-|{𝒲}_{ℋ}|$ such that $Z_{0}\in \Xi_{k(Z_{0})}$. In addition, let the trajectory from *Z*_0_ to ${𝒲}_{ℋ}$ as follows:


$$Z_{0}=\delta_{2^{2n}}^{i_{0}}\to \delta_{2^{2n}}^{i_{1}}\to \cdots \to \delta_{2^{2n}}^{i_{k(Z_{0})}}\in {𝒲}_{ℋ}.$$

Based on (18), for any state $\delta_{2^{2n}}^{i_{s}}$, $s\in [1:k(Z_{0})\;-\;1]$, there always exists a control $\delta_{2^{m}}^{l_{s}}\in \Delta_{2^{m}}$ such that $[F_{l_{s}}{]}_{i_{s+1},i_{s}}>0$. Then, let ${\text{Col}}_{i_{s}}(K)=\delta_{2^{m}}^{l_{s}}$, $s\in [1:k(Z_{0})\;-\;1]$. Due to the randomness of the initial state *Z*_0_, the logical matrix $K\in L_{2^{m}\times 2^{2n}}$ must satisfy equation (17). Therefore, based on Theorem 2, system (1) achieves asymptotic cluster synchronization with probability one. ◻

It is worth noting that according to Theorem 3 and its proof, using the sets $\Xi_{k}$, k∈ℕ, one can obtain the state feedback gain matrix *K* in system (6) that guarantees asymptotic cluster synchronization with probability one of (6). In addition, the design of matrix *K* is discussed below.

First, for all k∈ℕ, let

$$\Omega_{k}=\left\{j|\delta_{2^{2n}}^{j}\in (\Xi_{k}⧵\Xi_{k-1})\right\},$$ (20)

where $\Xi_{-1}=\emptyset$.

Then, according to the construction of sets $\Omega_{k}$, k∈ℕ, and Theorem 3, the following result can be obtained.

**Corollary 2.** *System (1) achieves asymptotic cluster synchronization with probability one if and only if the following conditions hold for system (9),*

*(i) set $\Omega_{0}$ is nonempty;*
*(ii) there exists a positive integer $T\leq 2^{2n}-|{𝒲}_{ℋ}|$, such that ${⋃}_{i=0}^{T}\Omega_{i}=[1:2^{2n}]$.*

Suppose the sets $\left\{\Omega_{k}|k\in [0:T]\right\}$ that satisfy the conditions in Corollary 2 is obtained. Then, the detailed construction of the state feedback gain matrix *K* for system (6) can be provided as follows:

$${\text{Col}}_{j}(K)\in \{\&\left\{\delta_{2^{m}}^{l}|\sum_{i\in \Omega_{0}}\left[(\delta_{2^{2n}}^{i}{)}^{⊤}{𝐅}_{l}\delta_{2^{2n}}^{j}\right]=1\right\},\&\&j\in \Omega_{0},\;\&\left\{\delta_{2^{m}}^{l}|\sum_{i\in \Omega_{k-1}}\left[(\delta_{2^{2n}}^{i}{)}^{⊤}{𝐅}_{l}\delta_{2^{2n}}^{j}\right]>0\right\},\&\&j\in \Omega_{k},\;k\in [1:T].$$ (21)

Based on Corollary 2, one can obtain that the system (1) achieves asymptotic cluster synchronization with probability one, if and only if there exists a state feedback controller of the form given by (21).

**Remark 1.** *The state feedback control design methodology in (21), derived from Corollary 2, can be extended to address finite-time cluster synchronization of system (1) by modifying the iterative procedure (18) as the following form, $\sum_{i\in \Xi_{k}}[{\left(\delta_{2^{2n}}^{i}\right)}^{⊤}(\sum_{l=1}^{2^{m}}F_{l})\delta_{2^{2n}}^{j}]=1$, for all k∈ℕ, with the initial condition $\Xi_{0}={𝒲}_{ℋ}$. This formulation ensures that for every state $X\in \Xi_{k+1}$, k∈ℕ, there exists a feedback control $\delta_{2^{m}}^{l}\in \Delta_{2^{m}}$ guaranteeing that state X converges to set $\Xi_{k}$ with probability one. Furthermore, while the proposed control strategy (21) guarantees minimal time cluster synchronization for arbitrary initial states in the finite-time case, it does not optimize the instantaneous synchronization probability at each time t for asymptotic synchronization scenarios.*

**Remark 2.** *State-flipping control, as an effective control methodology, has been widely applied in various fields, including BNs [43–45]. State-flipping control enables the modification of individual node states, thereby altering the state transition dynamics of BNs to achieve systems’ synchronization and stabilization. However, our work focuses on designing state feedback controllers that determine the inherent control input rules of the system without disrupting the internal topological structure or dynamic transition relationships between network nodes. For future studies, if the proposed equivalent algebraic conditions for cluster synchronization under DoS attacks cannot be satisfied, adopting state-flipping control to achieve cluster synchronization under DoS attacks would represent a feasible and meaningful research direction.*

### Double reinforcement learning method

In this subsection, we aim to propose a model-free RL algorithm to compute the state feedback gain matrix *K* in system (6), which ensures that system (6) achieves asymptotic cluster synchronization with probability one.

Next, we outline how the DDQN method can be applied to address the cluster synchronization problem of system (6). In light of Lemma 2 and Theorem 2, we will proceed to design an algorithm for system (9). System (9) is a Markov decision process (MDP) model. An MDP is an mathematical framework for sequential decision-making, represented by the tuple ⟨𝐒,𝐀,𝐏,𝐑⟩. Here, set **S** represents the set of all possible states of the environment, and $𝐒=\Delta_{2^{2n}}$ in system (9). Set **A** denotes the set of actions, and $𝐀=\Delta_{2^{m}}$ in system (9). The transition probability matrix **P** governs the rules for moving from the current state to the next state. In system (9), matrix 𝐏 is a column-stochastic matrix representing transition probabilities. Specifically, each entry ${𝐏}_{i,j}$ corresponds to the probability of transitioning to state $X_{t+1}=\delta_{2^{n}}^{j}$ from state $X_{t}=\delta_{2^{n}}^{j}$ under an action a(t) selected from the action set $𝐀=\Delta_{2^{n}}$. This is formally expressed as ${𝐏}_{i,j}=\mathrm{Pr}\{X_{t+1}=\delta_{2^{n}}^{j}|X_{t}=\delta_{2^{n}}^{j},a(t)\}$. It is worth noting that, during the RL process, the transition matrix **P** is unknown to the agent. The immediate reward received after executing action $\mu_{t}$ in state *X*_*t*_ is denoted by ${𝐑}_{X_{t},\mu_{t}}=𝔼\{r_{t+1}|X_{t},\mu_{t}\}$, where *r*_*t* + 1_ represents the immediate reward.

RL aims to select an optimal policy π:𝐒×𝐀→[0,1] that maximizes the expected sum of future rewards. The state-value function $v_{\pi }(X_{t})$ and the action-value function $q_{\pi }(X_{t},a_{t})$ are defined as follows:


$$v_{\pi }(X_{t})=\sum_{k=t+1}^{\infty }{𝔼}_{\pi }\{\gamma^{k-t-1}r_{k}|X_{t}\},\;\;q_{\pi }(X_{t},a_{t})=\sum_{k=t+1}^{\infty }{𝔼}_{\pi }\{\gamma^{k-t-1}r_{k}|X_{t},a_{t}\},$$

where γ is the discount factor, $X_{t}\in 𝐒$ is the state, and $a_{t}\in \Delta_{2^{m}}$ is the action. To find an optimal policy, the optimal action-value function q_*_(*x*,*a*) is defined as


$$q_{*}(X_{t},a_{t})=\max_{\pi }q_{\pi }(X_{t},a_{t}).$$

Furthermore, the optimal policy $\pi_{*}$ can be obtained by


$$\pi_{*}=\arg\max_{a\in \Delta_{2^{m}}}q_{*}(X_{t},a).$$

The optimal action-value function q_*_(*x*,*a*) is estimated as the following *Q*-function, which is updated using the Bellman equation,


$$Q(X_{t},a_{t})\leftarrow Q(X_{t},a_{t})+\alpha \left[r_{t+1}+\gamma \max_{a^{\prime }\in \Delta_{2^{m}}}Q(X_{t+1},a^{\prime })-Q(X_{t},a_{t})\right].$$

Here, $Q(X_{t},a_{t})$ is the expected reward for taking action *a*_*t*_ in state *X*_*t*_, *r*_*t* + 1_ and *X*_*t* + 1_ are the reward and state obtained after taking action *a*_*t*_, α is the learning rate, and γ is the discount factor. Additionally, $r_{t\;+\;1}\;+\;\gamma \max_{a^{\prime }\in \Delta_{2^{m}}}Q(X_{t+1},a^{\prime })$ is the TD target, and $r_{t+1}\;+\;\gamma \max_{a^{\prime }\in \Delta_{2^{m}}}Q(X_{t+1},a^{\prime })-Q(X_{t},a_{t})$ is the TD error.

Double-deep Q-learning (DDQN) is trained by minimizing the loss function defined as follows:


$$L(\omega )=\frac{1}{2}{\left(r_{t+1}+\gamma Q(X_{t+1},\mathrm{\backslash arg}\max_{a^{\prime }}Q(X_{t+1},a^{\prime };\omega );\omega^{-})-Q(X_{t},a_{t};\omega )\right)}^{2}.$$

The parameter ω is updated using stochastic gradient descent,


$$\omega \&=\omega -\alpha \nabla_{\omega }L(\omega )\;\&=\omega -\alpha (r_{t+1}+\gamma Q(X_{t+1},\arg\max_{a^{\prime }}Q(X_{t+1},a^{\prime };\omega );\omega^{-})\;\&\;-Q(X_{t},a_{t};\omega ))\nabla_{\omega }Q(X_{t},a_{t};\omega ).$$

To enhance the performance of DDQN, we employ the Prioritized Experience Replay (PER) technique [46] as the sampling method. Specifically, in the replay buffer ℬ, the probability of sampling any tuple $\left(X_{t},a_{t},r_{t+1},X_{t+1}\right)$ is defined as follows:


$$\mathrm{Pr}\{(X_{i},a_{i},r_{i+1},X_{i+1})\in ℬ\}=\frac{(p_{i}{)}^{\tau }}{\sum_{j\in ℬ}(p_{j}{)}^{\tau }},$$

where $p_{i}=|r_{i+1}+\gamma Q(X_{i+1},\mathrm{\backslash arg}\max_{a^{\prime }}Q(X_{i+1},a^{\prime };\omega );\omega^{-})-Q(X_{i},a_{i};\omega )|+\iota$, ι is a small constant, and ϖ controls the degree of prioritization.

However, prioritized replay may introduce bias. To address this, importance sampling weights are assigned as $\theta_{i}={\left(\frac{1}{\left|B|\cdot P(i\right)}\right)}^{\beta },$ where |ℬ| is the size of the replay buffer, $P(i)=Pr\{(X_{i},a_{i},r_{i+1},X_{i+1})\in ℬ\}$ is the probability of sampling the *i*-th experience, and β is a hyperparameter that gradually adjusts the degree of importance sampling during training to reduce bias while maintaining stability.

The state and action at time *t* are defined as *X*_*t*_ = *Z*(*t*) and *a*_*t*_ = *U*(*t*) for system (9), respectively, and a designed reward function is given by


$$r_{t}(X_{t},a_{t})=\left\{ \begin{array}{cc} 1, & X_{t}\in {𝒲}_{ℋ},\\ 0, & \text{otherwise}, \end{array}\right.$$

where ℋ is defined in (14).

Next, Algorithm 1 describes the setup and training of the DDQN algorithm incorporating PER.

**Algorithm 1. Cluster synchronization with probability one using DDQN.**



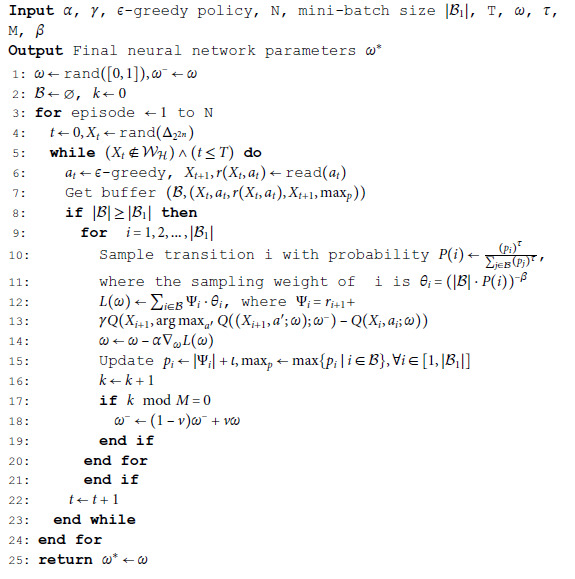



## Simulations

We consider a financial network comprising three investment institutions (A, B, and C) with coupled strategies in the renewable energy sector. Institution A operates as a subsidiary of institution B. The strategic alignment requirement between hierarchical entities necessitates that the investment strategies of A and B achieve asymptotic synchronization, while institution C, though organizationally independent from A and B, maintains bidirectional strategic interactions with them.

The investment decisions of these institutions are influenced by two external factors: government policy (*u*_1_), where *u*_1_ = 1 and *u*_1_ = 0 indicate favorable and unfavorable renewable energy policies, respectively; and bank loan interest rates (*u*_2_), where *u*_2_ = 1 and *u*_2_ = 0 represent interest rate reductions and increases, respectively.

To model the aforementioned financial network as a BCN, let $x_{i}(t)\in 𝒟$, i=1,2,3, denote the investment strategy of institutions A, B, and C at time t∈ℕ, respectively. Specifically, *x*_*i*_(*t*) = 1 and *x*_*i*_(*t*) = 0, i=1,2,3, represent the decisions of institutions A, B, and C to invest and not invest in the renewable energy sector, respectively.

However, the information exchange between institutions A and B is vulnerable to external adversarial attacks, such as DoS attacks, which can disrupt the exchange. This leads to non-real-time data reception between institutions, resulting in data loss. Therefore, let $y_{i}(t)\in 𝒟$, i=1,2,3, denote the observed strategy of institution A at time *t*, respectively. The data loss phenomenon follows a Bernoulli distribution, and the state observation of each institution at time *t* is expressed as


$$y_{i}(t)=\mu_{i}(t)x_{i}(t)+[1-\mu_{i}(t)]y_{i}(t-1),\;i=1,2,3.$$

Here, the sequence $\mu_{i}(t)$ is modeled as a Bernoulli distributed random variable with the following probability distribution,


$$\begin{array}{ccc} & \mathrm{Pr}\{\mu_{1}(t)=1\}=0.9, & \mathrm{Pr}\{\mu_{1}(t)=0\}=0.1;\\ & \mathrm{Pr}\{\mu_{2}(t)=1\}=0.8, & \mathrm{Pr}\{\mu_{2}(t)=0\}=0.2;\\ & \mathrm{Pr}\{\mu_{3}(t)=1\}=1, & \mathrm{Pr}\{\mu_{3}(t)=0\}=0. \end{array}$$

This suggests that 0.9, 0.8, and 1 represent the probabilities of successful information transmission for institutions A, B, and C, respectively.

Furthermore, assume that the strategic interactions evolve according to the following rules.

- Institution A withholds investment (*x*_1_(*t* + 1) = 0) if and only if both institutions B and C are observed in non-investment states ($y_{2}(t)=y_{3}(t)=0$).
- Institution B initiates investment (*x*_2_(*t* + 1) = 1) if both of the following hold:
  - a positive policy signal is present (*u*_1_(*t*) = 1), and
  - its subsidiary A is observed to be investing (*y*_1_(*t*) = 1).
- Institution C initiates investment (*x*_3_(*t* + 1) = 1) if either:
  - a positive policy incentive exists (*u*_1_(*t*) = 1), or
  - both of the following are true: interest rates are favorable (*u*_2_(*t*) = 1), and institution A is investing (*y*_1_(*t*) = 1).

Then, the strategic interactions can be modeled as a BCN under DoS atacks, described as follows:

$$\left\{ \begin{array}{c} x_{1}(t+1)=y_{2}(t)\vee y_{3}(t),\\ x_{2}(t+1)=u_{1}(t)\wedge y_{1}(t),\\ x_{3}(t+1)=u_{1}(t)\vee \left(u_{2}(t)\wedge y_{1}(t)\right). \end{array}\right.$$ (22)

**Fig 1:**
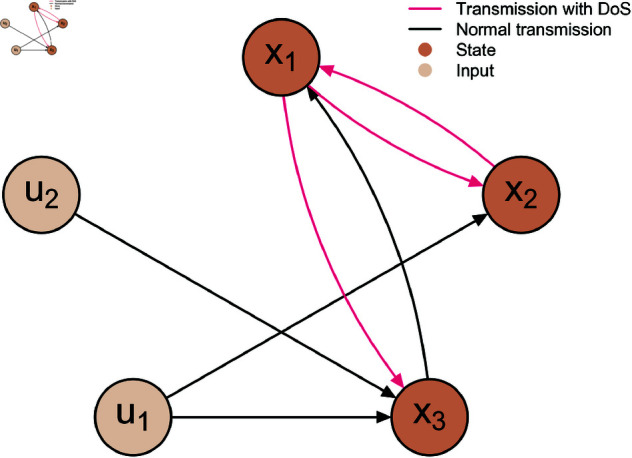
The network structure of system (22). Here, the directed edges colored in red represent information transmission processes between nodes that are subject to DoS attacks, resulting in probabilistic data loss, while the black directed edges indicate information transmission processes that are not subject to DoS attacks.

In addition, the network structure of system (22) is illustrated in Fig 1.

In order to obtain the state feedback control (7) that synchronizes the investment strategies between A (*x*_1_) and B (*x*_2_), let $X_{i}(t)=\left[\begin{array}{c} x_{i}(t)\\ \neg x_{i}(t) \end{array}\right]\in \Delta_{2}$, i=1,2,3, and $U_{j}(t)=\left[\begin{array}{c} u_{j}(t)\\ \neg u_{j}(t) \end{array}\right]\in \Delta_{2}$, j=1,2 in (22). Furthermore, let $X(t)={⋉}_{i=1}^{3}X_{i}(t)$ and $U(t)={⋉}_{j=1}^{2}U_{j}(t)$, then one has the following equivalent algebraic form of (22),

X(t+1)=LU(t)Y(t). (23)

Here, state transition matrix $L\in L_{8\times 32}$ is expressed as $L=[L_{1}\;L_{2}\;L_{3}\;L_{4}]$, where $L_{1}=L_{2}=\delta_{8}[1\;1\;1\;5\;3\;3\;3\;7]$, $L_{3}=\delta_{8}[3\;3\;3\;7\;4\;4\;4\;8]$, and $L_{4}=\delta_{8}[4\;4\;4\;8\;4\;4\;4\;8]$.

Since all nodes can be divided into two clusters $\Theta_{1}=\{1,2\}$ and $\Theta_{2}=\{3\}$, set ℋ defined in (14) can be obtained as ℋ={1,2,7,8}. Furthermore, the largest control invariant set of ℋ is $I_{c}(ℋ)=\{1,2,8\}$.

Then, let $Z(t)=X(t)⋉Y(t-1)\in \Delta_{64}$, based on (23) and the construction of system (9), one can obtain that

𝔼{Z(t+1)}=𝐅U(t)𝔼{Z(t)}. (24)

**Fig 2:**
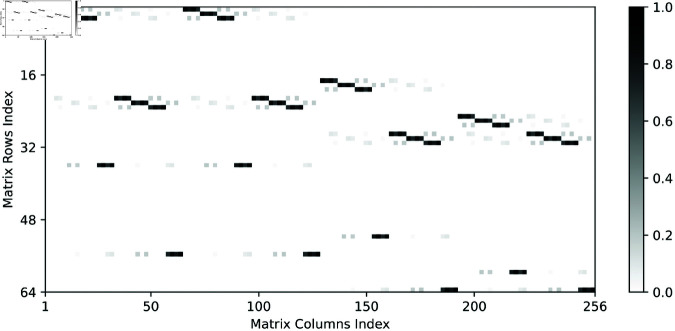
The state transition matrix of (24).

Here, the state transition matrix of (24), $𝐅\in P_{64\times 256}$, is constructed in Fig 2. Besides, ${𝒲}_{ℋ}=\{1,2,9,10,64\}$.

Based on the definition of sets $\Omega_{k}$, k∈ℕ, in (20), one can obtain that

$$\Omega_{0}=\&\{1,2,9,10,64\},\;\Omega_{1}=\&\{21,22,25,26\}\cup [1:18]\cup [29:36]\;\&\cup [41:44]\cup [47:50]\cup [57:64]\backslash \Omega_{0},\;\Omega_{2}=\&\{19:24,27,28,45,46\}\cup [37:40]\cup [51:56]\backslash \Omega_{0}\cup \Omega_{1}.$$ (25)

Furthermore, one has that U(t)=KX(t)Y(t−1), where

$${\text{Col}}_{j}(K)\in \{\&1,\&\&j\in [1:30]\cup [33:46]\cup [49:54],\;\&3,\&\&j\in [31:32]\cup [47:48]\cup [55:64].$$ (26)

Since $\cup_{i=0}^{2}\Omega_{i}=[1:64]$, based on Corollary 2, system (22) achieves asymptotic cluster synchronization with the state feedback gain matrix *K* defined by (26).

For instance, according to (26), for any time t∈ℕ, if institution A adopts an investment strategy (*x*_1_(*t*) = 1) while institutions B and C choose not to invest ($x_{2}(t)=x_{3}(t)=0$), and the observed strategies of all three institutions at time *t*–1 were non-investment ($y_{i}(t\;-\;1)=0,i=1,2,3$), we may implement the following external intervention measures at time *t*:

- No need for favorable renewable energy policies (*u*_1_(*t*) = 0),
- Reduced bank loan interest rates (*u*_2_(*t*) = 1).

**Fig 3:**
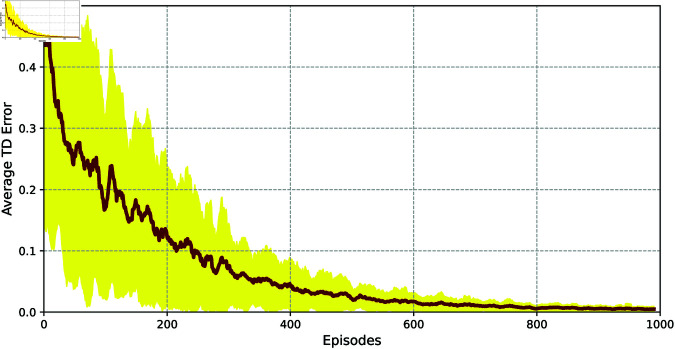
The average TD error across episodes.

**Fig 4:**
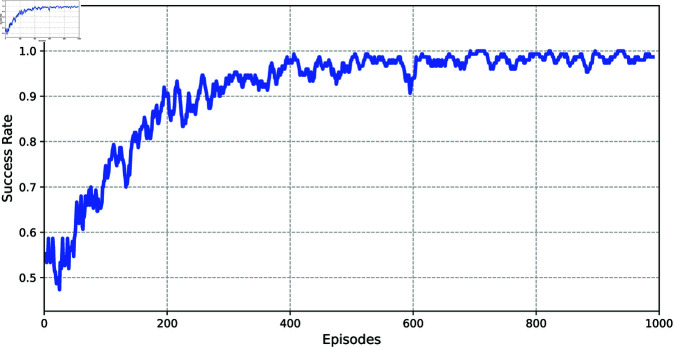
The average success rate across episodes.

Furthermore, let α=0.01, γ=0.99, *N* = 1000, |ℬ|=10000, $|{ℬ}_{1}|=64$, *T* = 5, β=0.4, and τ=0.6 in Algorithm 1. Then, Figs 3 and 4 show the TD error and success rate for reaching set ${𝒲}_{ℋ}$, averaged over 500 experimental episodes, respectively, demonstrating the effectiveness of our proposed method. Then, the state feedback gain matrix *K* of system (23) can be obtained below using the DDQN method,

$$K=\delta_{4}[\&1\;2\;1\;1\;4\;1\;2\;1\;2\;2\;4\;2\;2\;3\;3\;1\;1\;1\;1\;4\;4\;4\;1\;4\;4\;4\;4\;4\;4\;4\;4\;4\;\;\&4\;1\;4\;4\;4\;4\;2\;3\;2\;3\;4\;4\;4\;4\;2\;4\;1\;3\;4\;4\;1\;4\;4\;4\;4\;2\;3\;4\;4\;4\;4\;4].$$ (27)

**Fig 5:**
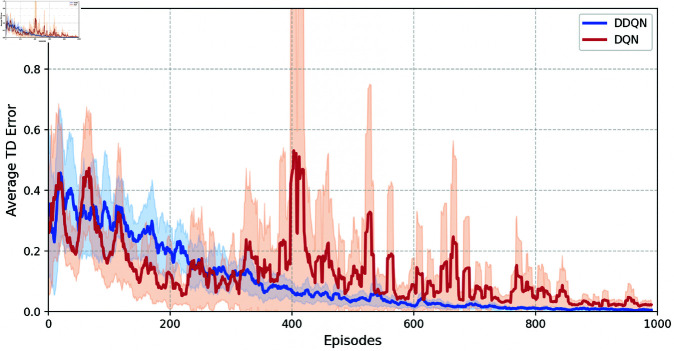
The average TD error across episodes of DDQN and DQN methods.

**Fig 6:**
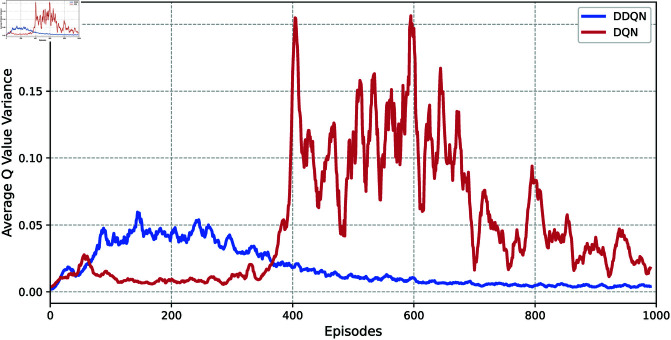
The average Q value variance across episodes of DDQN and DQN methods.

In order to demonstrate the advantages of the DDQN method, let α=0.01, γ=0.99, *N* = 1000, |ℬ|=500, $|{ℬ}_{1}|=16$, *T* = 5, β=0.4, and τ=0.6 in Algorithm 1. Then, Figs 5 and 6 show the TD error and variance of Q value for reaching set ${𝒲}_{ℋ}$ of the DDQN and deep Q-learning (DQN) mathods, respectively. We can observe that under this parameter configuration, DDQN method exhibits significantly faster convergence than DQN method, along with lower Q-value variance. This demonstrates the stability and effectiveness of the DDQN method.

For instance, according to (27), for any time t∈ℕ, if all institutions A, B, and C adopts an investment strategy (*x*_*i*_(*t*) = 1,*i* = 1,2,3), and the observed strategies of all three institutions at time *t*–1 were investment ($y_{i}(t\;-\;1)=1,i=1,2,3$), we may implement the following external intervention measures at time *t*:

- Implement favorable renewable energy policies (*u*_1_(*t*) = 1),
- Reduced bank loan interest rates (*u*_2_(*t*) = 1).

## Conclusion

This paper has investigated the asymptotic cluster synchronization of BCNs under DoS attacks, where each state node has been subject to random data loss governed by a Bernoulli distribution. The algebraic representation of BCNs under DoS attacks has been established through the STP method, enabling a systematic analysis framework. Necessary and sufficient algebraic conditions for achieving asymptotic cluster synchronization under DoS attacks have been derived. For scenarios with known and unknown system models, suitable state feedback controllers ensuring asymptotic cluster synchronization have been successfully designed. The set-iteration method has addressed the model-based case, while a DDQN approach has been developed for the model-free case. Furthermore, a DDQN algorithm has been designed to identify an appropriate state feedback control policy. The efficacy of these methods has been validated through a numerical simulation, demonstrating robust cluster synchronization under DoS attacks.

## References

[1] Kauffman SA. Metabolic stability and epigenesis in randomly constructed genetic nets. J Theor Biol. 1969;22(3):437–67. doi: 10.1016/0022-5193(69)90015-0 5803332

[2] Huang C, Wang W, Lu J, Kurths J. Asymptotic stability of boolean networks with multiple missing data. IEEE Trans Automat Contr. 2021;66(12):6093–9. doi: 10.1109/tac.2021.3060733

[3] Wang Y, Li B, Pan Q, Zhong J, Li N. Asymptotic synchronization in coupled Boolean and probabilistic Boolean networks with delays. Nonl Anal: Hybrid Syst. 2025;55:101552. doi: 10.1016/j.nahs.2024.101552

[4] Liu F, Sun Y, Zhang C, Xu L, Zhang H. Set stability and synchronization of generalized asynchronous probabilistic Boolean networks with impulsive effects. PLoS One. 2025;20(2):e0318038. doi: 10.1371/journal.pone.0318038 39937801 PMC11819517

[5] Eduati F, Corradin A, Di Camillo B, Toffolo G. A Boolean approach to linear prediction for signaling network modeling. PLoS One. 2010;5(9):e12789. doi: 10.1371/journal.pone.0012789 20862273 PMC2940821

[6] Rivera-Torres P, Llanes Santiago O. Fault detection and isolation in smart grid devices using probabilistic boolean networks. Computational intelligence in emerging technologies for engineering applications. 2020. p. 165–85.

[7] Akutsu T, Hayashida M, Ching W-K, Ng MK. Control of Boolean networks: hardness results and algorithms for tree structured networks. J Theor Biol. 2007;244(4):670–9. doi: 10.1016/j.jtbi.2006.09.023 17069859

[8] Cheng D, Qi H, Li Z. Analysis and control of Boolean networks: a semi-tensor product approach. Springer. 2010.

[9] Wang Y, Zhong J, Pan Q, Li N. Minimal pinning control for set stability of Boolean networks. Appl Math Comput. 2024;465:128433. doi: 10.1016/j.amc.2023.128433

[10] Chen H, Wang Z, Shen B, Liang J. Model evaluation of the stochastic boolean control networks. IEEE Trans Automat Contr. 2022;67(8):4146–53. doi: 10.1109/tac.2021.3106896

[11] Chen H, Wang Z, Liang J, Li M. State estimation for stochastic time-varying boolean networks. IEEE Trans Automat Contr. 2020;65(12):5480–7. doi: 10.1109/tac.2020.2973817

[12] Chen H, Wang Z, Shen B, Liang J. Distributed recursive filtering over sensor networks with nonlogarithmic sensor resolution. IEEE Trans Automat Contr. 2022;67(10):5408–15. doi: 10.1109/tac.2021.3115473

[13] Li Y, Li H, Ding X. Set stability of switched delayed logical networks with application to finite-field consensus. Automatica. 2020;113:108768. doi: 10.1016/j.automatica.2019.108768

[14] Ma Z, Wang ZJ, McKeown MJ. Probabilistic Boolean network analysis of brain connectivity in Parkinson’s disease. IEEE J Sel Top Signal Process. 2008;2(6):975–85. doi: 10.1109/jstsp.2008.2007816

[15] Kabir MH, Hoque MR, Koo B-J, Yang S-H. Mathematical modelling of a context-aware system based on Boolean control networks for smart home. In: The 18th IEEE International Symposium on Consumer Electronics (ISCE 2014). 2014. p. 1–2. doi: 10.1109/isce.2014.6884406

[16] Diveev AI, Sofronova EA. Synthesis of intelligent control of traffic flows in urban roads based on the logical network operator method. In: 2013 European Control Conference (ECC), 2013. p. 3512–7. doi: 10.23919/ecc.2013.6669696

[17] Roli A, Villani M, Serra R, Benedettini S, Pinciroli C, Birattari M. Dynamical properties of artificially evolved Boolean network robots. In: AI*IA 2015 Advances in Artificial Intelligence; 2015. p. 45–57.

[18] Makarov SI, Boldyrev MA. Application of bool variables in analysis of risks in the bond market. Digital Technologies in the New Socio-Economic Reality. 2022. p. 479–88.

[19] Boccaletti S, Latora V, Moreno Y, Chavez M, Hwang D. Complex networks: structure and dynamics. Phys Rep. 2006;424(4–5):175–308. doi: 10.1016/j.physrep.2005.10.009

[20] Allen F, Gale D. Optimal currency crises. Carnegie-Rochester Conf Ser Publ Policy. 2000;53(1):177–230. doi: 10.1016/s0167-2231(01)00030-6

[21] Xu X, Zeng Z, Xu J, Zhang M. Fuzzy dynamical system scenario simulation-based cross-border financial contagion analysis: a perspective from international capital flows. IEEE Trans Fuzzy Syst. 2017;25(2):439–59. doi: 10.1109/tfuzz.2016.2574928

[22] Zhao Z, Chen D, Wang L, Han C. Credit risk diffusion in supply chain finance: a complex networks perspective. Sustainability. 2018;10(12):4608. doi: 10.3390/su10124608

[23] Feng S, Cetinkaya A, Ishii H, Tesi P, Persis CD. Networked control under DoS attacks: tradeoffs between resilience and data rate. IEEE Trans Automat Contr. 2021;66(1):460–7. doi: 10.1109/tac.2020.2981083

[24] Zhu S, Lu J, Cao J, Lin L, Lam J, Ng M, et al. Undetectable attacks on Boolean networks. In: 2023 62nd IEEE Conference on Decision and Control (CDC). 2023. p. 1698–703. doi: 10.1109/cdc49753.2023.10383321

[25] Wang Y-W, Zeng Z-H, Liu X-K, Liu Z-W. Input-to-state stability of switched linear systems with unstabilizable modes under DoS attacks. Automatica. 2022;146:110607. doi: 10.1016/j.automatica.2022.110607

[26] Mölsä J. Mitigating denial of service attacks: a tutorial. JCS. 2005;13(6):807–37. doi: 10.3233/jcs-2005-13601

[27] Abdollahi Biron Z, Dey S, Pisu P. Real-time detection and estimation of denial of service attack in connected vehicle systems. IEEE Trans Intell Transport Syst. 2018;19(12):3893–902. doi: 10.1109/tits.2018.2791484

[28] Falowo OI, Ozer M, Li C, Abdo JB. Evolving malware and DDoS attacks: decadal longitudinal study. IEEE Access. 2024;12:39221–37. doi: 10.1109/access.2024.3376682

[29] Kochemazov S, Semenov A. Using synchronous Boolean networks to model several phenomena of collective behavior. PLoS One. 2014;9(12):e115156. doi: 10.1371/journal.pone.0115156 25526612 PMC4272282

[30] Gualdi S, Cimini G, Primicerio K, Di Clemente R, Challet D. Statistically validated network of portfolio overlaps and systemic risk. Sci Rep. 2016;6:39467. doi: 10.1038/srep39467 28000764 PMC5175158

[31] Musciotto F, Marotta L, Piilo J, Mantegna RN. Long-term ecology of investors in a financial market. Palgrave Commun. 2018;4(1). doi: 10.1057/s41599-018-0145-1

[32] Mu T, Feng J-E, Wang B, Jia Y. Identification of Boolean control networks with time delay. ISA Trans. 2024;144:113–23. doi: 10.1016/j.isatra.2023.10.016 37865590

[33] Van Hasselt H, Guez A, Silver D. Deep reinforcement learning with double Q-learning. AAAI. 2016;30(1):1–7. doi: 10.1609/aaai.v30i1.10295

[34] Acernese A, Yerudkar A, Glielmo L, Vecchio CD. Double deep-Q learning-based output tracking of probabilistic boolean control networks. IEEE Access. 2020;8:199254–65. doi: 10.1109/access.2020.3035152

[35] Moschoyiannis S, Chatzaroulas E, Šliogeris V, Wu Y. Deep reinforcement learning for stabilization of large-scale probabilistic Boolean networks. IEEE Trans Control Netw Syst. 2023;10(3):1412–23. doi: 10.1109/tcns.2022.3232527

[36] Brewer J. Kronecker products and matrix calculus in system theory. IEEE Trans Circuits Syst. 1978;25(9):772–81. doi: 10.1109/tcs.1978.1084534

[37] Mehmood S, Amin R, Mustafa J, Hussain M, Alsubaei FS, Zakaria MD. Distributed Denial of Services (DDoS) attack detection in SDN using optimizer-equipped CNN-MLP. PLoS One. 2025;20(1):e0312425. doi: 10.1371/journal.pone.0312425 39869573 PMC11771897

[38] Salim MM, Rathore S, Park JH. Distributed denial of service attacks and its defenses in IoT: a survey. J Supercomput. 2019;76(7):5320–63. doi: 10.1007/s11227-019-02945-z

[39] Patil S, Chaudhari S. DoS attack prevention technique in wireless sensor networks. Procedia Comput Sci. 2016;79:715–21. doi: 10.1016/j.procs.2016.03.094

[40] Salem FM, Youssef H, Ali I, Haggag A. A variable-trust threshold-based approach for DDOS attack mitigation in software defined networks. PLoS One. 2022;17(8):e0273681. doi: 10.1371/journal.pone.0273681 36037194 PMC9423637

[41] Ren Y, Lu J, Liu Y, Shi K. Cluster synchronization of Boolean networks under probabilistic function perturbation. IEEE Trans Circuits Syst II. 2022;69(2):504–8. doi: 10.1109/tcsii.2021.3086985

[42] Guo Y, Zhou R, Wu Y, Gui W, Yang C. Stability and set stability in distribution of probabilistic Boolean networks. IEEE Trans Automat Control. 2018;64(2):736–42.

[43] Ni J, Tang Y, Li F. Minimum-cost state-flipped control for reachability of Boolean control networks using reinforcement learning. IEEE Trans Cybern. 2024;54(11):7103–15. doi: 10.1109/TCYB.2024.3454253 39288054

[44] Du L, Zhang Z, Xia C. A state-flipped approach to complete synchronization of Boolean networks. Appl Math Comput. 2023;443:127788. doi: 10.1016/j.amc.2022.127788

[45] Du L, Zhang Z, Xia C. A node-pinning and state-flipped approach to partial synchronization of Boolean networks. Nonl Anal: Hybrid Syst. 2024;53:101501. doi: 10.1016/j.nahs.2024.101501

[46] Schaul T, Quan J, Antonoglou I, Silver D. Prioritized experience replay. International Conference on Learning Representations. 2016; p. 1–23.

